# Primary Bile Acid Diarrhea: A Narrative Review of Pathophysiology, Diagnostic Challenges, and Emerging Therapeutic Strategies

**DOI:** 10.7759/cureus.108847

**Published:** 2026-05-14

**Authors:** Ayisha Maqsood, Syeda Kashaf Fatima, Sanjeev Kumar, Monia Mohosina Moni, Majd Aldin Oudeh, Mohammad Ghaith Hulo, Rameeqa Ejaz, Alaa Abdelfattah, Rana Muhammad Naveed, Syed Muhammad Ali

**Affiliations:** 1 Medicine, Allied Hospital Faisalabad, Faisalabad, PAK; 2 Internal Medicine, Foothills Medical Centre, Calgary, CAN; 3 Biochemistry, Indira Gandhi Institute of Medical Sciences, Patna, IND; 4 Health Sciences, Cardiff Metropolitan University, Cardiff, GBR; 5 Medicine, Department of Health, Abu Dhabi, ARE; 6 Medicine, Odessa National Medical University, Odessa, UKR; 7 Acute Medicine, Pilgrim Hospital, Boston, GBR; 8 Medicine, Ayub Medical College, Abbottabad, PAK; 9 Medicine, Dubai Medical University, Dubai, ARE; 10 Medicine, Bahawal Victoria Hospital, Bahawalpur, PAK; 11 Surgery, Weill Cornell Medicine - Qatar, Doha, QAT; 12 Acute Care Surgery, Hamad General Hospital, Doha, QAT

**Keywords:** bile acid sequestrants, chronic watery diarrhea, diagnostic gap, fgf19, ibs-d misdiagnosis, idiopathic bile acid malabsorption, primary bile acid diarrhea, sehcat, serum c4, underdiagnosis

## Abstract

Primary bile acid diarrhea (BAD) is a clinically relevant cause of persistent watery diarrhea caused by high bile acid production and poor enterohepatic feedback regulation. A growing body of evidence suggests that disrupting the farnesoid X receptor-fibroblast growth factor 19 (FXR-FGF19) signaling pathway contributes to aberrant bile acid metabolism and increased colonic output. Despite increased understanding of its link with diarrhea-predominant irritable bowel syndrome (IBS-D), primary BAD remains difficult to diagnose due to overlapping clinical symptoms and a scarcity of specialist diagnostic tools. This narrative review highlights current data on the pathogenesis, clinical presentation, diagnostic modalities, and evolving therapy approaches for primary BAD, with a focus on existing diagnostic limitations and unmet clinical needs. A thorough literature search was conducted using electronic databases such as PubMed, Scopus, Embase, and Google Scholar. BAD, bile acid malabsorption, IBS-D, FXR, FGF19, SeHCAT, serum C4, fecal bile acids, and bile acid sequestrants were all covered using both Medical Subject Headings (MeSH) and free-text phrases. A narrative synthesis was conducted on pertinent original research, reviews, clinical trials, and guidelines published in English. According to available data, a major mechanism underlying primary BAD is defective FXR-FGF19-mediated feedback inhibition. Nonspecific symptoms, overlap with functional gastrointestinal illnesses, and limited access to established diagnostic instruments, such as the SeHCAT retention scan, are the causes of persistent diagnostic difficulties. Alternative biomarkers, such as fecal bile acid measurement, FGF19, and blood 7α-hydroxy-4-cholesten-3-one (C4), show promise for diagnosis but are still unreliable and poorly standardized. The mainstay of treatment remains bile acid sequestrants, although there are no uniform management algorithms, and treatment response varies. Future therapy options may be enhanced by novel strategies targeting bile acid signaling pathways.

## Introduction and background

Chronic diarrhea is one of the most common and challenging presentations encountered in gastroenterology, affecting nearly 5% of the population worldwide [1-3]. Diarrhea that does not resolve can significantly lower the quality of life by restricting daily activities and creating a great deal of psychological and physical distress. Bile acid diarrhea (BAD), especially in its primary or idiopathic form, remains a significant yet often disregarded cause among its several etiologies. While secondary BAD develops as a result of illnesses such as ileal disease, bowel resection, or cholecystectomy, primary BAD arises in the absence of recognizable structural gastrointestinal disease, ileal resection, or prior biliary surgery [1-3]. Despite increasing recognition of the disorder, primary BAD continues to be underdiagnosed because its symptoms closely resemble those of functional gastrointestinal disorders, especially irritable bowel syndrome with diarrhea (IBS-D).

The liver produces bile acids, which typically help break down and absorb lipids from food. The majority of bile acids are recycled through the enterohepatic circulation after being reabsorbed in the terminal ileum under physiological conditions. To avoid too much bile acid buildup in the colon, this process is strictly controlled. This regulatory mechanism is compromised in primary BAD, leading to elevated bile acid production and excessive colonic bile acid delivery. Overproduction of colonic bile acids increases intestinal motility and fluid secretion, which, in turn, causes loose stools, urgency, bloating, and increased stool frequency [4-6].

Growing research over the last 20 years has shown that a significant percentage of people who were previously diagnosed with functional diarrhea or IBS-D may actually have primary BAD. When properly examined, studies indicate that approximately 25-30% of patients with persistent functional diarrhea symptoms may actually have underlying BAD [4]. Additionally, about 25% of individuals meeting Rome III criteria for IBS-D have been shown to have idiopathic BAD, underscoring its clinical relevance and frequent misdiagnosis [4]. All of these results point to primary BAD as a moderately common but underdiagnosed condition affecting 1-2% of the general population [1-4].

Recent findings have identified disrupted feedback control of bile acid synthesis as a key mechanism in the pathophysiology of BAD. Fibroblast growth factor 19 (FGF19), an intestinal hormone that inhibits further bile acid synthesis in the liver by activating the farnesoid X receptor (FXR) signaling pathway, is normally produced when bile acid is absorbed in the ileum. Excessive hepatic bile acid production can result from decreased FGF19 synthesis or impaired signaling through related receptors, such as fibroblast growth factor receptor 4 and β-klotho [4-6]. As a result, excess bile acids reach the colon and activate receptors, including Takeda G-protein-coupled receptor 5 (TGR5), thereby accelerating colonic transit and promoting intestinal secretion, contributing to symptoms of diarrhea [5,6].

Primary BAD remains significantly underdiagnosed in routine clinical practice, despite advances in mechanistic knowledge. Several variables, including symptom overlap with IBS-D, low clinician awareness, and limited availability of conclusive diagnostic tests, cause this diagnosis gap. The selenium homocholic acid taurine (SeHCAT) retention test is still unavailable or underutilized in many healthcare settings, despite being the gold standard for diagnosis in many areas [7,8]. Although other biomarkers, such as fasting serum C4 and FGF19 levels, have demonstrated diagnostic potential, their routine clinical use remains uneven [7,8]. Before BAD is finally taken into consideration, many people endure protracted symptoms, several investigations, a delayed diagnosis, and ineffective treatment.

This review summarizes current evidence regarding primary idiopathic BAD in patients without ileal disease or prior cholecystectomy. It examines the epidemiology and underlying pathophysiologic mechanisms of the disorder, evaluates existing diagnostic strategies and their limitations, and explores factors contributing to persistent underdiagnosis and misclassification. By consolidating current knowledge, this review aims to improve clinical recognition of primary BAD and highlight practical approaches for reducing diagnostic delays and improving patient outcomes.

## Review

This narrative review provides a detailed synthesis of current evidence on primary (idiopathic) BAD, focusing on pathophysiology, diagnostic challenges, and management strategies.

Methodology

Literature Search Strategy

A structured literature search was performed using PubMed/MEDLINE, Embase, Scopus, Web of Science, and Google Scholar. The search covered literature from January 2000 to December 2025. Search terms included combinations of “bile acid diarrhea,” “primary bile acid malabsorption,” “idiopathic BAD,” “chronic watery diarrhea,” “IBS-D,” “SeHCAT,” “FGF19,” “C4 biomarker,” and “bile acid sequestrants.” Reference lists of relevant articles were also manually screened to identify additional studies. Controlled trials, systematic reviews, and meta-analyses were considered. Included studies involved adults or children with primary BAD (no ileal disease or resection), focusing on epidemiology, mechanisms, diagnostics, or treatment. Editorials, opinions, and single case reports with limited relevance were not included. Non-English-language publications and studies without full-text access were also excluded.

Study Selection and Synthesis

Two reviewers independently reviewed studies for relevance and quality. Evidence was narratively synthesized with a focus on high-quality, recent studies. Mechanistic studies were included if relevant to the FXR-FGF19 pathway. Two authors also independently assessed the studies for clarity of reporting, methodology, and design. During synthesis, studies with major problems or high risk of bias were given lower priority. This careful review process aimed to increase the rigor, credibility, and clarity of the review.

Pathophysiological basis of disease: dysregulation of the farnesoid X receptor-fibroblast growth factor 19 axis and its impact on bile acid homeostasis

The enterohepatic circulation of bile acids is tightly controlled to maintain balance. About 95% of bile acids are secreted into the duodenum. The process is tightly regulated by the apical sodium-dependent bile acid transporter. This triggers the FXR in ileal enterocytes, leading to the release of FGF19. FGF19 enters the portal blood and acts on the liver. There, it inhibits the enzyme cholesterol 7α-hydroxylase (CYP7A1), which controls bile acid production. This feedback helps maintain bile acid levels. If the process is disrupted, bile acid production runs unchecked, leading to primary BAD [9,10]. According to population-based and referral cohort studies, primary BAD accounts for 25-35% of cases previously diagnosed with IBS-D [11,12]. A meta-analysis found that about one-third of IBS-D patients had objective evidence of bile acid malabsorption [13]. This overlap is clinically relevant, as these patients frequently do not respond to conventional IBS treatments. Although all ages can be affected, middle-aged adults are most frequently diagnosed. There is a slight female predominance [14]. Geographic variation in reported prevalence mainly reflects diagnostic availability, not true epidemiologic differences. There is a significant disease burden. Patients’ quality of life and productivity at work are negatively impacted by crippling urgency, frequent bowel movements, and fear of incontinence [15]. Before a proper diagnosis is made, the patient often sees multiple specialists, undergoes unnecessary endoscopic procedures, and faces prolonged empirical treatments. This increases healthcare costs [16].

Epidemiological distribution of primary bile acid diarrhea

**Figure 1:**
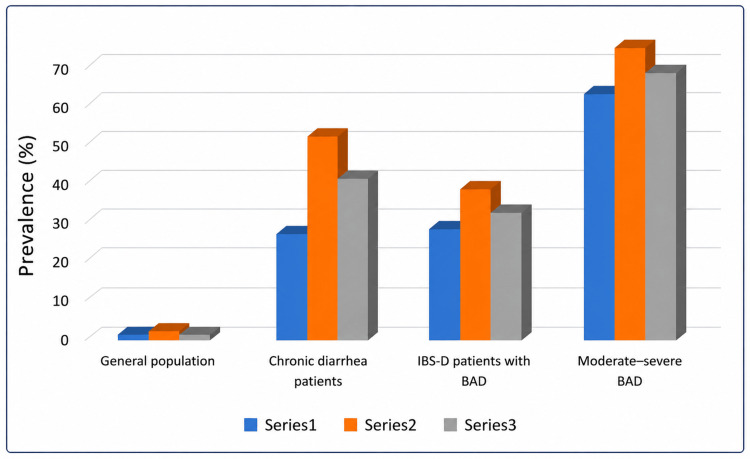
Epidemiology of primary bile acid diarrhea. Epidemiological distribution of primary BAD across patient subgroups. The largest proportions (50%, 60%) represent overlapping or co-occurring risk factors (e.g., idiopathic BAD and post-cholecystectomy). In comparison, intermediate (20%) and minor (10% each) fractions correspond to less prevalent associations (e.g., Crohn's disease, microscopic colitis, and genetic variants). Cumulative percentages exceeding 100% reflect disease heterogeneity and concurrent etiologies, emphasizing the multifactorial nature of primary BAD. Series 1 represents diagnostic assessment by the SeHCAT test, Series 2 represents serum-based biomarkers, and Series 3 represents serum FGF19 levels. The figure was created by the authors using Excel and is not AI-generated. BAD = bile acid diarrhea; SeHCAT = selenium homocholic acid taurine; FGF19 = fibroblast growth factor 19

Primary BAD demonstrates a variable yet clinically significant distribution across different populations. In the general population, its prevalence is estimated at approximately 1-2%, although this is likely underestimated due to limited diagnostic recognition [16,17]. Among patients presenting with chronic unexplained diarrhea, the prevalence increases markedly, ranging from 25% to 50%, underscoring its importance as a major yet underdiagnosed etiology in gastroenterology practice. Notably, within individuals diagnosed with IBS-D, approximately 25-35% are found to have underlying bile acid malabsorption, highlighting substantial diagnostic overlap. Furthermore, among confirmed cases of BAD, 60-70% exhibit moderate-to-severe disease based on objective measures such as SeHCAT retention values, indicating that clinically significant disease predominates. Collectively, these data emphasize that primary BAD is not only common but also frequently misclassified, reinforcing the need for improved diagnostic strategies and clinical awareness [16,17] (Figure 1).

Advancing the diagnosis of bile acid diarrhea: comparative insights into SeHCAT, serum biomarkers, and fecal bile acid assessment

The diagnosis of primary BAD remains challenging due to the lack of a universally accessible gold-standard test. A critical appraisal of available diagnostic modalities highlights significant gaps in current practice (Table 1) [18-31].

**Table 1:** Comparison of diagnostic modalities for primary bile acid diarrhea. This table summarizes the key diagnostic tools used to evaluate primary BAD, including the SeHCAT retention test, serum biomarkers (C4 and FGF-19), fecal bile acid quantification, and empirical BAS trials. It highlights their underlying principles, diagnostic performance, advantages, limitations, and real-world availability, emphasizing the challenges in establishing a standardized diagnostic approach across different healthcare settings. BAD = bile acid diarrhea; SeHCAT = selenium homocholic acid taurine; FGF19 = fibroblast growth factor 19; BAS = bile acid sequestrant

Modality	Principle	Key advantages	Key limitations	Sensitivity/Specificity	Availability
SeHCAT retention test [18,24,31]	Measures the 7-day retention of radiolabeled synthetic bile acid	Gold standard; objective quantification; high diagnostic accuracy	Requires nuclear medicine, radiation exposure and limited availability	Sensitivity: 85–95%, specificity: 85–100% [18,24,31]	Very low (mostly Europe)
Serum C4 (7α-hydroxy-4-cholesten-3-one) [26,30,31]	Marker of hepatic bile acid synthesis (CYP7A1 activity)	A simple blood test reflects bile acid overproduction	Diurnal variation; assay variability; lack of standard cut-offs	Sensitivity: 70–90%, specificity: 70–85% [26,30,31]	Moderate
Serum FGF19 [26,29,30]	Marker of FXR-mediated ileal feedback inhibition	Reflects gut–liver axis dysfunction	Inter-assay variability; no standardized thresholds	Sensitivity: 60–80%, specificity: 65–85% [26,29,30]	Low
Fecal bile acids (48-hour collection) [22,31]	Direct measurement of bile acid excretion in stool	Direct physiological assessment differentiates primary vs. secondary	Inconvenient collection; dietary/transit variability	Sensitivity: 70–90%, specificity: 75–90% [22,31]	Very low
Empirical BAS trial [14,27,24]	Symptomatic response to bile acid binding agents	Widely available; low cost; therapeutic–diagnostic approach	Subjective response, placebo effect, lack of standardization	Sensitivity 60–80%, low specificity [14,27,24]	High

**Figure 2:**
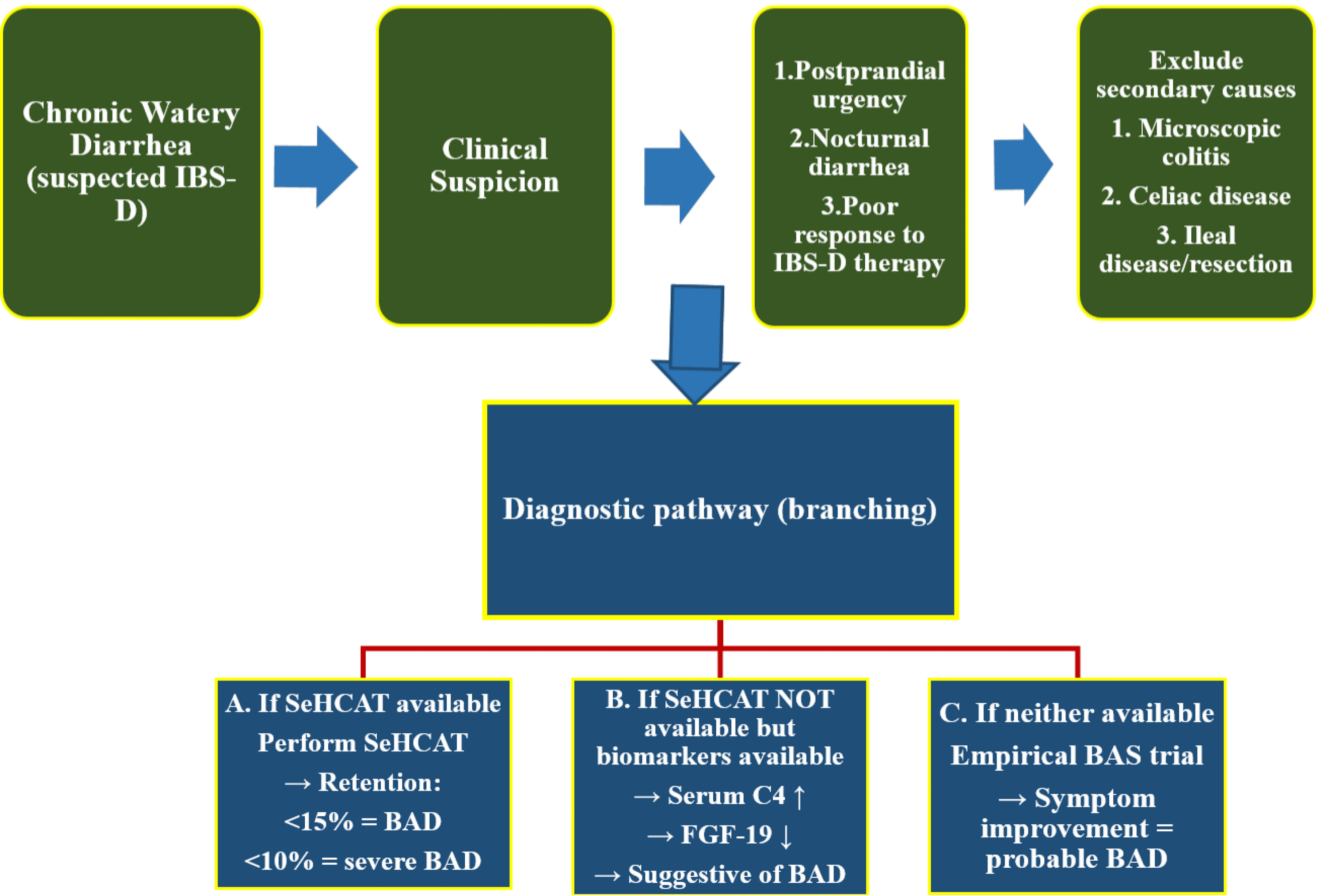
Stepwise diagnostic algorithm for primary bile acid diarrhea: a resource-based approach. This schematic illustrates a stepwise diagnostic approach to primary BAD, beginning with clinical suspicion and exclusion of secondary causes. The pathway then adapts to resource availability, utilizing SeHCAT as the gold standard, serum biomarkers (C4 and FGF-19), when available, or an empirical bile acid sequestrant trial in limited settings to establish diagnosis. The diagnostic approach is aligned with current expert recommendations and published evidence [4,7,18]. The figure was created by the authors using Word and is not AI-generated. BAD = bile acid diarrhea; SeHCAT = selenium homocholic acid taurine; FGF19 = fibroblast growth factor 19

A practical diagnostic procedure is crucial for clinical practice, given the limitations of SeHCAT and biomarkers. Based on clinical indicators, including postprandial urgency, nocturnal symptoms, and poor response to conventional IBS-D medications, the algorithm starts with a high index of suspicion. The diagnostic approach diverges based on test availability after conventional investigations have ruled out secondary causes (such as celiac disease, microscopic colitis, and structural ileal disease). SeHCAT is the recommended confirmatory test when available; retention values below 10% indicate severe disease, and below 15% indicate BAD. Elevated fasting serum C4 and decreased FGF-19 together provide compelling evidence when SeHCAT is unavailable, but serum indicators are available. An empirical trial of a bile acid sequestrant (BAS) is a practical and acceptable approach for both diagnosis and treatment in resource-constrained settings where neither SeHCAT nor biomarkers are available. While adjusting the workup to the resources at hand, this methodical strategy enables doctors to manage diagnostic uncertainty, as shown in Figure 2.

Clinical presentation and diagnostic clues in bile acid diarrhea: a pattern-based approach to early recognition

Any patient who presents with persistent watery diarrhea should be suspected of having primary BAD, especially if they have typical clinical symptoms. Frequent, urgent, loose, or watery stools are commonly reported by patients and often occur soon after meals. Nocturnal diarrhea may help differentiate functional problems from BAD. Abdominal pain, bloating, and fecal incontinence are possible additional symptoms, especially in cases of extreme urgency [19,20]. A key diagnostic clue is the poor or absent response to conventional IBS-D therapies, such as fiber supplementation, antispasmodics, or loperamide. On the other hand, even when used experimentally, a partial or full response to BAS substantially confirms the diagnosis. IBS-D, microscopic colitis, celiac disease, small intestinal bacterial overgrowth, and pancreatic exocrine insufficiency are among the many potential diagnoses. Nocturnal symptoms, postprandial aggravation, and positive objective findings are distinguishing features that support a diagnosis of BAD [21], as shown in Table 2.

**Table 2:** Clinical features and differential diagnosis of primary bile acid diarrhea. This table outlines the characteristic clinical presentation of primary BAD, including symptom patterns, treatment response, and distinguishing features, alongside key differential diagnoses such as IBS-D, microscopic colitis, celiac disease, SIBO, and pancreatic exocrine insufficiency. It provides a pattern-based framework to aid early clinical recognition and differentiation from other causes of chronic diarrhea. BAD = bile acid diarrhea; IBS-D = irritable bowel syndrome with diarrhea; SIBO = small intestinal bacterial overgrowth

Feature	Clues suggestive of primary BAD	Key differential diagnoses
Symptoms	Chronic watery diarrhea, often postprandial; urgency; nocturnal diarrhea; fecal incontinence; bloating and abdominal discomfort [19,20]	IBS-D: typically lacks nocturnal diarrhea and objective biochemical abnormalities
Treatment response	Poor or absent response to conventional IBS-D therapies (fiber, loperamide, antispasmodics); often shows partial or marked response to bile acid sequestrants (e.g., cholestyramine) [20,21]	Microscopic colitis: may respond to budesonide; confirmed by colonic biopsy showing lymphocytic or collagenous inflammation
Risk factors	Often idiopathic (primary BAD); slight female predominance; middle-aged adults; may overlap with IBS-D population [19]	Celiac disease: associated with weight loss, iron deficiency anemia, positive serology (tTG-IgA), and villous atrophy on biopsy
Distinguishing features/Investigations	Objective evidence of bile acid malabsorption: elevated serum C4, reduced FGF19 levels, or abnormal SeHCAT retention (<10–15%) where available [20,21]. Symptom improvement with bile acid sequestrants supports diagnosis	SIBO: positive breath tests (glucose/lactulose hydrogen), prominent bloating; responds to antibiotics
-	-	Pancreatic exocrine insufficiency: steatorrhea, weight loss, low fecal elastase-1, and fat malabsorption

Current management strategies

BAS, which lower intestinal exposure to excess bile acids, are the mainstay of treatment for primary BAD. The most researched medication, cholestyramine, successfully binds intraluminal bile acids and relieves symptoms in about 70-80% of patients with proven BAD. However, poor palatability, bloating, constipation, and medication interactions frequently restrict its use. Colesevelam, a more recent BAS pill, has better tolerability and fewer drug interactions, but its price may prevent some people from using it. To strike a balance between tolerance and efficacy, careful dose titration is necessary. By lowering bile acid stimulation, dietary modification, specifically a low-fat diet (≤40-50 g/day) and smaller, more frequent meals, acts as a helpful adjuvant. By reestablishing feedback inhibition of bile acid synthesis, emerging targeted treatments such as FXR agonists (e.g., obeticholic acid) and FGF19 analogs aim to address the underlying pathology. These medicines are still in the exploratory stage and are not yet used in routine clinical practice, despite early research showing promise [22], as shown in Table 3.

**Table 3:** Management strategies for primary bile acid diarrhea. This table presents the therapeutic approaches for primary bile acid diarrhea (BAD), including first-line bile acid sequestrants (cholestyramine, colesevelam, colestipol), adjunctive dietary modifications, and emerging targeted therapies such as FXR agonists and FGF-19 analogues. Key considerations regarding efficacy, tolerability, mechanism of action, and clinical applicability are highlighted to guide individualized patient management. The table has been prepared manually and compiled using Microsoft Word. BAD = bile acid diarrhea; FXR = farnesoid X receptor; FGF19 = fibroblast growth factor 19

Strategy	Agents/Intervention	Key considerations
First-line: Bile acid sequestrants	Cholestyramine (powder), colesevelam (tablet), colestipol	Cholestyramine: Effective but poor palatability, drug interactions. Colesevelam: Better tolerated, tablet formulation, fewer drug interactions, but more expensive. Titrate dose to balance efficacy and side effects (constipation, bloating)
Adjunctive: Dietary modification	Low-fat diet (≤4,050 g/day); smaller, more frequent meals	Reduces bile acid stimulation and load. Safe, low-risk supportive measure
Investigational: Targeted therapies	FXR agonists (e.g., obeticholic acid); FGF19 analogues; microbiome-based therapies	Emerging agents that target underlying pathophysiology. Currently limited to clinical trials. Long-term safety/efficacy unknown

Clinical implications and unmet needs

The underdiagnosis of primary BAD carries significant clinical and economic consequences. Missed diagnoses lead to persistent, debilitating symptoms that adversely affect quality of life, work productivity, and psychological well-being. Chronic, unexplained diarrhea often leads to repeated consultations, unnecessary diagnostic procedures, and ineffective treatments, thereby increasing healthcare costs.

On the other hand, a precise diagnosis enables focused BAS treatment, which often leads to rapid symptom relief and reduced healthcare utilization [23]. This emphasizes the importance of greater clinical knowledge and more organized diagnostic methods. Even with increased awareness, significant gaps remain. Given that SeHCAT is restricted to specific areas, the absence of a widely available gold-standard diagnostic test continues to impede diagnosis [24]. Furthermore, the clinical relevance of biomarkers such as blood C4 and FGF19 is limited by the lack of standardized tests and well-recognized cutoff values [25]. Heterogeneous practice patterns stem from the lack of guidance in current clinical guidelines for stepwise screening for BAD in patients with chronic diarrhea [26]. Additionally, there is a dearth of prospective, population-based data on the natural history, genetic risk, and optimal treatment of primary idiopathic BAD, as most current research has focused on secondary BAD [27]. Lastly, despite the effectiveness of BAS, tolerability problems continue, underscoring the need for mechanism-based therapies that are better tolerated [28]. Future research should prioritize developing and validating accessible, point-of-care diagnostic tools, including rapid serum or fecal assays, to overcome geographic and economic barriers. Standardization of C4 and FGF19 testing, along with the establishment of universally accepted cutoff values, is essential for broader clinical implementation [29]. To assess the efficacy and cost-effectiveness of diagnostic algorithms that incorporate clinical features, biomarkers, and empirical treatment trials, pragmatic clinical trials are required. Disseminating streamlined diagnostic routes in primary care and general gastroenterology settings should be the main goal of implementation strategies. Furthermore, the use of genetic, metabolomic, and microbiome data in precision medicine can predict therapeutic response and enable tailored treatment [30,31]. These developments could turn primary BAD, a poorly understood illness, into a well-defined and successfully treated disorder.

However, primary bile acid diarrhea is an underappreciated but significant contributor to the worldwide burden of chronic gastrointestinal illness from the standpoint of public health. Considerable diagnostic misclassification and unmet needs in regular care are highlighted by population-level studies that indicate a considerable percentage of patients classified as IBS-D may really have underlying bile acid malabsorption [32,33]. In primary care settings, when diagnostic resources are scarce, this mistake increases healthcare utilization, prolongs morbidity, and lowers quality-adjusted life years [32,33]. Additionally, discrepancies in care delivery across healthcare systems are caused by the differential availability of diagnostic modalities, such as SeHCAT scanning, leading to inequitable diagnostic and management outcomes between high-resource and low-resource settings [32]. Improving BAD detection could prevent unnecessary long-term IBS medication, reduce repetitive diagnostic testing, and increase the effectiveness of gastroenterologist service allocation at the system level [33]. As a result, BAD should be viewed as a modifiable public health burden with consequences for healthcare fairness and efficiency in addition to a clinical diagnostic difficulty [32,33].

## Conclusions

IBS-D is sometimes mistaken for primary idiopathic BAD, a common but underdiagnosed and highly curable illness. It results in increased exposure to colonic bile acid and secretory diarrhea due to disruption of the FXR-FGF19 feedback loop. Lack of well-defined biomarkers, inadequate clinical awareness, and limited access to gold-standard diagnostic tests are the main reasons for its underdiagnosis. These gaps must be filled, as an organized diagnostic strategy that accounts for clinical characteristics, accessible biomarkers, and empirical studies of BAS can greatly enhance patient outcomes, reduce morbidity, and reduce unnecessary medical use. To further serve this underprivileged patient community, future research should focus on developing easily accessible diagnostic tools, validating biomarkers, and devising tailored treatment approaches.
